# Treatment with the arginase inhibitor N_w_-hydroxy-nor-L-arginine restores endothelial function in rat adjuvant-induced arthritis

**DOI:** 10.1186/ar3860

**Published:** 2012-05-30

**Authors:** Clément Prati, Alain Berthelot, Bernadette Kantelip, Daniel Wendling, Céline Demougeot

**Affiliations:** 1 EA 4267 Fonctions et Dysfonctions epithéliales, University of Franche Comté, 19 rue Ambroise Paré, 25030 Besançon, France; 2 Department of Rheumatology, University Hospital Jean Minjoz, 3 bd Fleming, 25000 Besançon, France; 3 Department of Pathologic Anatomy, University Hospital Jean Minjoz, 3 bd Fleming, 25000 Besançon, France; 4 EA 4266 Agents pathogènes et inflammation, University of Franche Comté, 2 place St Jacques, 2503 Besançon, France

## Abstract

**Introduction:**

Endothelial dysfunction (ED) participates to atherogenesis associated to rheumatoid arthritis. We recently reported increased arginase activity/expression in vessels from adjuvant-induced arthritis (AIA) rats. In the present study, we investigated the effects of a curative treatment with the arginase inhibitor N_w_-hydroxy-nor-L-arginine (nor-NOHA) on vascular dysfunction in AIA rats.

**Methods:**

AIA rats were treated with nor-NOHA (40 mg/kg/d, ip) for 21 days after the onset of arthritis. A group of untreated AIA rats and a group of healthy rats served as controls. ED was assessed by the vasodilatory effect of acetylcholine (Ach) on aortic rings. The role of superoxide anions, prostanoids, endothelium-derived hyperpolarizing factor (EDHF) and nitric oxide synthase (NOS) pathway was studied. Plasma levels of IL-6 and vascular endothelial growth factor (VEGF) were determined by ELISA kits. Arthritis severity was estimated by a clinical, radiological and histological analysis.

**Results:**

Nor-NOHA treatment fully restored the aortic response to Ach to that of healthy controls. The results showed that this beneficial effect is mediated by an increase in NOS activity and EDHF and reduced superoxide anion production as well as a decrease in the activity of cyclooxygenase (COX)-2, thromboxane and prostacyclins synthases. In addition, nor-NOHA decreased IL-6 and VEGF plasma levels in AIA rats. By contrast, the treatment did not modify arthritis severity in AIA rats.

**Conclusions:**

The treatment with an arginase inhibitor has a potent effect on ED in AIA independently of the severity of the disease. Our results suggest that this new pharmacological approach has the potential as a novel add-on therapy in the treatment of RA.

## Introduction

Rheumatoid arthritis (RA) is a chronic systemic inflammatory disease characterized by articular and extra-articular manifestations involving cardiovascular diseases, which accounts for 30 to 50% of all deaths [1]. Recent studies showed that atherosclerosis lesions occur earlier and have a more rapid evolution in RA patients than in the general population [1]. Endothelial dysfunction is thought to be a key event in the development of atherosclerosis and has been identified in patients with RA, even in the early diagnosed arthritis [1]. It is generally defined as impaired endothelium-dependent vasodilation to specific stimuli and characterized by an imbalance between vasoconstriction and vasodilation factors. Although the improvement of endothelial function is recognized as an important element of the global management of RA patients [2], the precise pathophysiological mechanisms of endothelial dysfunction in RA are still poorly understood.

Consistent with the findings in humans, a few studies reported impaired endothelial function in the model of adjuvant-induced arthritis (AIA) in rats. In this model, endothelial dysfunction positively correlates with disease activity [3]. However, data concerning the pathophysiological features of endothelial dysfunction are scarce. Previous studies reported that vessels from AIA rats exhibited a deficiency in tetrahydrobiopterin (BH4), the co-factor of nitric oxide synthase (NOS) [4] and overproduced superoxide anions (O_2_^-.^) [4-6]. Surprisingly, whether there is an impairment of the production of endothelium-derived vasodilator factors, such as nitric oxide (NO), prostacyclin (PGI_2_) and endothelium-derived hyperpolarizing factor (EDHF) or of contractile factors such as angiotensin-II (ANG-II), endothelin-1 (ET-1) and thromboxane A_2 _(TXA_2_) is not known. Recently, we identified the vascular arginase upregulation as a new interesting mechanism contributing to endothelial dysfunction in AIA rats [3].

Arginase (EC 3.5.3.1) is a hydrolytic enzyme responsible for converting L-arginine to L-ornithine and urea. Mammalian arginases exist in two distinct isoforms (type I and type II), which have specific subcellular localizations and tissues distribution. Notably, both arginase isoforms are expressed by endothelial and vascular smooth muscle cells [7]. Because NOS and arginase use L-arginine as a common substrate, arginase may downregulate NO biosynthesis by competing with NOS for L-arginine degradation. Consistent with this hypothesis, increased vascular arginase activity was reported in various animal models of cardiovascular diseases [8,9] and a few studies demonstrated the benefits of a chronic treatment with an arginase inhibitor for treating endothelial dysfunction associated to hypertension [3,10,11], diabetes [12], atherosclerosis [13] or ageing [14]. These pharmacological data have been partly confirmed by the data obtained from the mouse strains with genetic ablation of arginase expression. Although mice lacking arginase I (Arg I ^-/-^) die in the perinatal period as a consequence of a non-functional urea cycle [15], mice with homologous deletion of arginase II expression (Arg II ^-/-^) are viable, have high plasma levels of arginine and exhibit an enhanced vasorelaxation to acetylcholine [16]. In the AIA model, we found that increased arginase activity/expression correlated with arthritis severity [3]. Moreover, our data suggested, at least *in vitro*, that the upregulation of arginase contributes to endothelial dysfunction likely by limiting the L-arginine availability for NOS [3]. However, whether the treatment with an arginase inhibitor may constitute a good strategy for RA-associated endothelial dysfunction is not known.

Besides the vasculature, a few studies investigated the occurrence of arginase pathway abnormalities at the articular level in RA and have yielded controversial results. One study reported decreased arginase activity in the synovial fluid of RA patients [17], whereas other studies demonstrated increased arginase activity and expression in the synovial fluid [18] as well as in plasma [19]. Again, the pathophysiological role of arginase at the articular level is still unknown, and whether arginase inhibition might modify the severity of the disease has never been investigated.

The aim of the present study was to determine the effects of a curative treatment with N_w_-hydroxy-nor-L-arginine (nor-NOHA), a selective arginase inhibitor, in AIA rats. The effect of nor-NOHA on vascular reactivity to vasodilating and vasoconstrictive drugs was evaluated on aortic rings, with special emphasis on the mechanisms involved in endothelial dysfunction. Additionally, we assessed the impact of the treatment on clinical, radiological and histological articular parameters as well as on peripheral markers of inflammation and endothelial function.

## Materials and methods

### Induction of AIA, clinical evaluation and treatment

A total of 48 male Lewis rats were purchased from Janvier (Le Genest Saint Isle, France). Animals were kept under a 12 h:12 h light:dark cycle and allowed free access to food and water. The investigation conforms with the Guide for the Care and Use of Laboratory Animal published by the US National Institutes of Health (NIH publication No. 85-23, revised 1996) and was approved by our local animal ethics committee.

Adjuvant arthritis was induced by a single intradermal injection at the base of the tail of 1 mg of heat-killed *Mycobacterium butyricum *(Difco, Detroit, MI, USA) suspended in 0.1 ml of mineral oil (Freund's incomplete adjuvant (Difco). The animals developed arthritis between Day 10 and Day 15. At the first symptoms of arthritis, arthritic rats were randomly divided into two groups: untreated AIA rats (*n *= 20) and nor-NOHA-treated AIA rats (*n *= 20). Nor-NOHA (Bachem, France) was dissolved in saline and administered once a day (40 mg/kg, ip) for 21 consecutive days. This inhibitor was chosen because it is a potent selective arginase inhibitor without interfering with NOS activity [20] or L-arginine uptake [21]. Nor-NOHA inhibited arginase activity in aorta with an IC50 of less than 1 μM, that is, similar to its effects on liver arginase (IC50 = 0.5 μM). We previously demonstrated that the dose of 40 mg/kg/day was well-tolerated and led to reduction of arginase I activity (liver isoform, -41%) and arginase II activity (kidney isoform, -40%) [11], without any modification of plasma urea levels [10]. Untreated AIA received an equal volume of saline daily. With this protocol, the treatment was performed during the secondary chronic phase of AIA, that is, during the development of severe disease and during the phase at which systemic inflammatory markers are high [22-24]. A group of untreated non-arthritic age-matched rats was used as controls (*n *= 8).

Rats were weighed and monitored seven days per week in a blinded fashion for clinical signs of arthritis. The scoring system was employed as follows [25]: arthritis of one finger scores 0.1, weak and moderate arthritis of one big joint (ankle or wrist) scores 0.5 and intense arthritis of one big joint scores 1. Tarsus and ankle was considered as the same joint. Sum of joints scores of four limbs leads to an arthritic score of maximum 6 to each rat. The ankle diameter was measured with a digital caliper (Vernier Stainless, Guangxi, China).

### Tissue preparation

Twenty-one days after the onset of arthritis, rats were anaesthetized with pentobarbital (60 mg/kg, ip). Blood was withdrawn from the abdominal aorta, immediately centrifuged at 4,000 *g *for 10 minutes, and plasma was stored at -80°C until analysis. Thoracic aortas were removed, cleaned and immediately used for vascular reactivity studies. Ankles were removed and placed in 4% formalin.

### Vascular reactivity

Arthritis was induced in five rats per week so that the onset of arthritis was shifted by one week for the different groups. For one given rat, studies were performed on both endothelium intact and endothelium denuded rings, five days a week. For the experiments without incubation of an inhibitor, the same ring was used for different experiments. With this protocol, the study of aortic vasodilator response was conducted over a 10-week period. The thoracic aorta was excised, cleaned of connective tissue and cut into rings of approximately 2 mm in length. Rings were suspended in Krebs solution (NaCl 118 mmoles/liter, KCl 4.65 mmoles/liter, CaCl_2 _2.5 mmoles/liter, KH_2_PO_4 _1.18 mmoles/liter, NaHCO_3 _24.9 mmoles/liter, MgSO_4 _1.18 mmoles/liter, glucose 12 mmoles/liter, pH 7.4), maintained at 37°C and continuously aerated with 95% O_2_, 5% CO_2_, for isometric tension recording in organs chambers, as previously described [26]. In some rings, endothelium was mechanically removed. The completeness of endothelial denudation was confirmed by the absence of relaxation to the endothelium-dependent agonist acetylcholine (Ach, 10^-6 ^moles/liter). After a 90-minute-equilibration period under a resting tension of 2 g, rings with intact endothelium were constricted with norepinephrine (NE, 3. 10^-7 ^moles/liter) and endothelium-dependent relaxation was assessed with Ach (10^-11^-10^-4 ^moles/liter). To dissect the mechanisms of endothelial dysfunction, rings were incubated for 30 minutes respectively with the non-selective NO synthase inhibitor N_W_-nitro-L-arginine methyl ester (L-NAME, 10^-4 ^moles/liter), the selective inhibitor of inducible NOS (1400 W, 10^-5 ^moles/liter), the Ca^2+^-dependent K^+ ^channels inhibitors apamin (10^-7 ^moles/liter) and charybdotoxin (10^-7 ^moles/liter), the non-selective COX inhibitor indometacin (10^-5 ^moles/liter), the selective COX-2 inhibitor (NS398, 10^-5 ^moles/liter), the prostacyclin (PGI_2_) synthase inhibitor tranylcypromine (10^-5 ^moles/liter), the thromboxane (TX) synthase inhibitor furegrelate (10^-6 ^moles/liter), the superoxide dismutase mimetic (SOD) Tempol (10^-4 ^moles/liter) and with the add of "respectively" above we must let "and" the nicotinamide adenine dinucleotide phosphate (NADPH) oxidase inhibitor apocynin (3. 10^-4 ^moles/liter). To determine the sensitivity of vascular smooth muscle cells to physiological vasoconstrictive factors, endothelium-denuded rings were constricted with NE (10^-11^-10^-4 ^moles/liter), ET-1 (10^-10^-10^-6 ^moles/liter) or ANG-II (10^-10^-10^-6 ^moles/liter). Finally, we evaluated the endothelium-independent relaxation to the NO donor sodium nitroprussiate (SNP, 10^-11^-10^-4 ^moles/liter) after constriction with NE 3. 10^-7 ^moles/liter.

### Plasma IL-6, IL-1β, TNFα and VEGF levels

Plasma levels of interleukin-6 (IL-6), interleukin-1β (IL-1β) and tumor necrosis factor-alpha (TNFα), three peripheral markers of inflammation and plasma concentration of the vascular endothelial growth factor (VEGF), a peripheral marker of endothelial dysfunction in many cardiovascular diseases [27,28], were determined by using enzyme-linked immunosorbent assay (ELISA) kits according to the manufacturer's instructions (Bender MedSystems, Vienna, Austria). The limits of detection of ELISA kits provided by the manufacturer for TNF α, IL-6, IL-1β and VEGF were 11.2 pg/mL, 12 pg/mL, 4 pg/mL and 27 pg/mL, respectively.

### Radiographical *ex vivo *analysis of joints of ankle and foot

The high resolution digital X-rays (25 kV, 10 mA) of hind paws of rats in three groups were performed with a Nova Siemens Mammomat 3000 (Erlangen, Germany) the city is Erlangen at Day 21 after the onset of inflammatory joint signs. A score of 0 to 3 was determined for each paw, according to Du *et al. *[29]. This score takes into account the swelling of soft parts, the thickness of the joint space, bone destruction and the neoformation of periosteal tissue, as follows: 0 = normal, 1 = soft tissue swelling only, 2 = soft tissue swelling and early erosions and 3 = severe erosions, the maximum score for each rat being 6.

### Histological analysis

Ankles were decalcified and embedded in paraffin and sections of 6 μm were performed and stained with hematoxylin-eosin and safranin (HES). The histological features of cartilage, bone and periarticular soft tissue were evaluated in a blind fashion by using the score of Kinne *et al. *[30]. According to this score, the degree of inflammatory infiltration is scored from 0 to 3 where 0 = no infiltration, 1 = slight infiltration, 2 = moderate infiltration and 3 = severe infiltration. The degree of bone and cartilage destruction is scored from 0 to 4, where 0 = no erosion of cartilage or bone, 1 = unequivocal erosion ≤ 10% of cartilage or bone trabeculae, 2 = erosions ≤ 50% 3 = erosions from 50 to 90% and 4 = erosions > 90%. The maximum score per rat is 7.

### Data and statistical analysis

Values are presented as the mean ± SEM. The values of maximal relaxation (Emax values) were determined by fitting the original dose-response curves using the Sigma Plot program (Systat Software, Chicago, IL USA). The curves obtained from aortic rings were compared by two-way analysis of variance. Comparison between groups was assessed by one-way analysis of variance (ANOVA) followed by Bonferroni's test. The analysis of the relationship between two parameters was determined by linear regression analysis and Spearman's correlation coefficient was calculated between these variables. *P *< 0.05 was considered statistically significant.

## Results

### Arthritis severity was unaffected by nor-NOHA treatment

**Figure 1:**
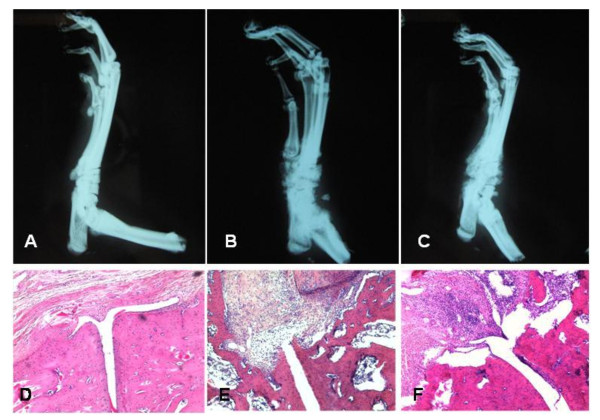
**Radiographs and histological aspect of hind limbs at Day 21 after the onset of arthritis**. Experiments were performed in controls rats (**A, D**), AIA rats (**B, E**) and nor-NOHA treated rats (**C, F**). On radiographs, both AIA and treated AIA rats exhibited a thickening of the soft tissues of the ankle and foot as well as a joint destruction and periosteal tissue neoformation. Histological slices were stained with HES (enlargement × 20). A severe joint destruction associated to synovial proliferation was present in AIA and treated AIA rats.

Body weights, ankle diameters and arthritis scores are shown in Table 1. Compared to control rats, AIA rats exhibited a significantly reduced body weight and a higher ankle diameter. The nor-NOHA treatment did not affect body weight, ankle diameter or arthritic score in AIA rats. Nor-NOHA did not modify the time-course of arthritic scores and did not delay the occurrence of arthritis symptoms, as presented in more details in Additional file 1. The radiological and histological analysis of AIA rats showed bone and cartilage erosions with joint space narrowing, inflammatory synovitis and inflammatory infiltration of the soft parts that were unaffected by nor-NOHA (Figure 1). Accordingly, the histological and radiological scores of nor-NOHA-treated AIA rats were not different from those of untreated AIA rats (Table 1).

**Table 1:** Clinical, histological and radiological scores and plasma levels of IL-6 and VEGF

	control rats (*n *= 8)	AIA rats (*n *= 20)	nor-NOHA treated rats (*n *= 20)
Body weight (g)	310 ± 3	279 ± 10*	269 ± 5*
Arthritis score	0	1.3 ± 0.2*	1.1 ± 0.2*
Ankle diameter (mm)	7.6 ± 0.1	8.3 ± 0.2*	8.3 ± 0.1*
Histological score	0	4.1 ±	3.7 ± 1*
Radiological score	0	2.2 ±	2.1 ± 0.6*
IL-6 (pg/ml)	366 ± 22	419 ± 17*	378 ± 22
VEGF (pg/ml)	488 ± 36	642 ± 34*	500 ± 65

Data were obtained 21 days after the onset of arthritis in control rats, AIA rats and nor-NOHA treated rats. Values are the mean ± SEM. * = *P <*0.05 vs control

### Nor-NOHA treatment decreased IL-6 and VEGF plasma levels in AIA rats

TNFα and IL-1β were not detectable in plasma whatever the group of rats (data not shown). IL-6 and VEGF plasma levels were significantly higher in AIA rats as compared to control rats (*P *< 0.05, Table 1). Nor-NOHA significantly prevented the increase in IL-6 and VEGF levels in AIA rats. A significant positive correlation was found between VEGF and endothelial dysfunction as attested by the negative correlation identified between the Emax of Ach and the plasma VEGF levels (r = - 0.536, *P *= 0.007). Additional file 2 shows this in more detail. By contrast, IL-6 levels did not correlate with Emax of Ach (r = 0.001, *P *= 0.959, data not shown).

### Nor-NOHA treatment improved vascular function in AIA rats

**Figure 2:**
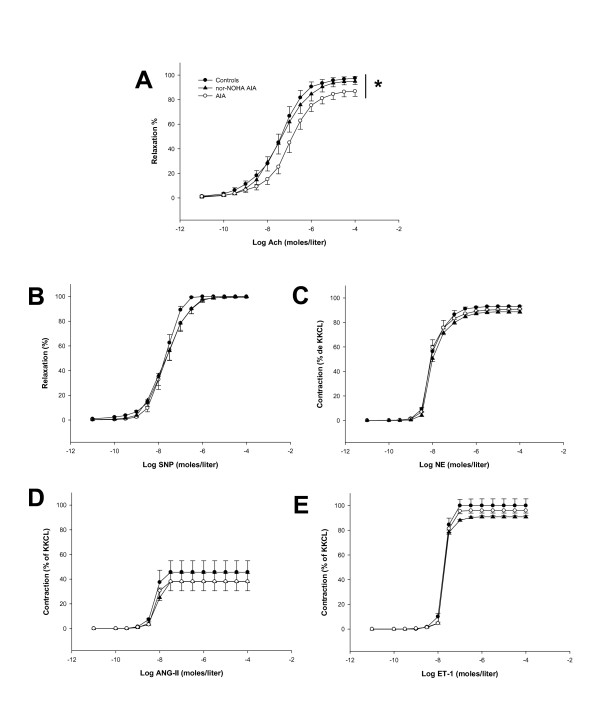
**Vascular reactivity to vasodilators and vasoconstrictive agents**. Experiments were performed on thoracic aortic rings from control rats, AIA rats and nor-NOHA AIA rats 21 days after the onset of arthritis. **A**, Concentration - response curve for Ach in endothelium-intact rings preconstricted with NE 3 × 10^-7 ^moles/liter. **B**, Concentration - response curve for SNP in endothelium-denuded rings preconstricted with NE 3 × 10^-7 ^moles/liter. **C**, Concentration - response curve for NE in endothelium-denuded rings, **D**, Concentration - response curve for ANG-II in endothelium-denuded rings, **E**, Concentration - response curve for ET-1 in endothelium-denuded rings. Values are the mean ± SEM from 8 to 12 rings. * = *P *< 0.05. KKCl = Krebs potassium chloride solution.

To confirm the presence of endothelial dysfunction in AIA rats, we first investigated the vasodilatory response of endothelium-intact rings to Ach. As shown in Figure 2A, as a reflection of endothelial dysfunction, the relaxant effect of Ach was significantly reduced in AIA rats compared to control rats. Importantly, nor-NOHA treatment totally reversed the impairment of Ach-induced relaxation in AIA rats (Figure 2A). By contrast, no difference in SNP-induced vasorelaxation was observed among control rats, untreated AIA and nor-NOHA-treated AIA rats (Figure 2B), thus confirming the normality of the response of VSMC to exogenous NO, as previously reported [3]. To determine whether aortic rings from AIA rats exhibited an altered response to physiologically-relevant vasoconstrictors, the effect of NE, ANG-II and ET-1 was evaluated. As shown in Figures 2C-E, the vasoconstrictive responses to NE, ANG-II and ET-1 were not different between the three groups of rats. Overall results indicate that the impaired Ach-induced vasorelaxation observed in AIA rats strictly resulted from an impairment of endothelial production of relaxant factors.

**Figure 3:**
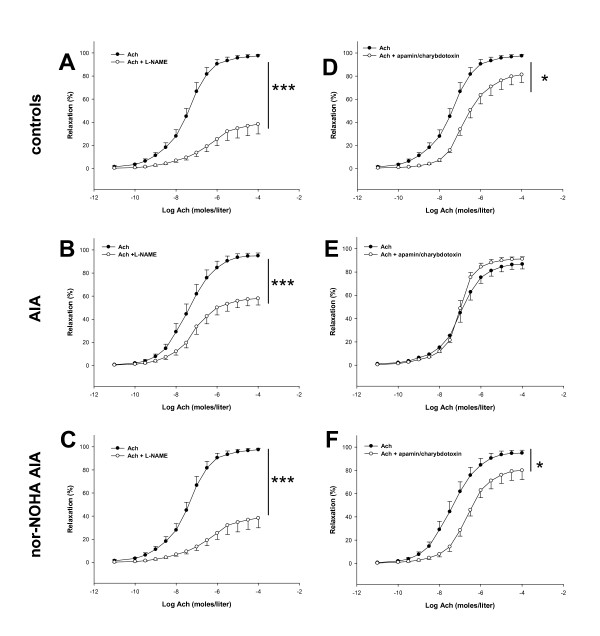
**Effect of L-NAME and the combination of apamin/charybdotoxin on vasodilation response to Ach**. Experiments were performed on aortic rings isolated from control rats (**A, D**), AIA rats (**B, E**) and nor-NOHA AIA rats (**C, F**) 21 days after the onset of arthritis. Cumulative concentration-responses curves with Ach were obtained after a 60-minute incubation period with L-NAME at 10^-4 ^moles/liter (A, B, C) or with apamin-charybdotoxin at 10^-7 ^moles/liter each (D, E, F). Values are the mean ± SEM from 8 to 12 rings. * = *P *< 0.05, *** = *P *< 0.001.

**Figure 4:**
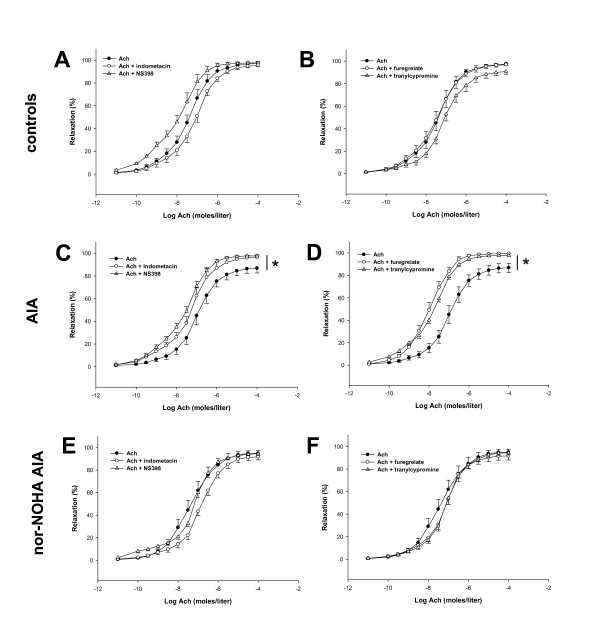
**Effect of indometacin, NS398, furegrelate and tranylcypromin on vasodilation response to Ach**. Cumulative concentration curves were obtained in aortic rings isolated from AIA, nor-NOHA AIA and control rats 21 days after the onset of arthritis. Cumulative concentration curves with Ach were obtained after a 60-minute incubation period with indometacin at 10^-5 ^moles/liter or with NS398 at 10^-4 ^moles/liter in controls (**A**), AIA (**C**) and nor-NOHA AIA (**E**) or with furegrelate at 10^-6 ^moles/liter or with tranylcypromine at 10^-5 ^moles/liter in controls (**B**), AIA (**D**) and nor-NOHA AIA (**F**). Values are the mean ± SEM from 8 to 12 aortic rings. * = *P <*0.05.

To determine the mechanisms involved in the beneficial effects of nor-NOHA treatment on endothelial function, we first investigated the contribution of the NOS and EDHF pathways in Ach-induced vasorelaxation in control and AIA rats. As shown in Figure 3, as a reflection of a NOS-activation after Ach challenge, L-NAME, a non-selective NOS inhibitor, significantly reduced the Ach-dependent relaxation in all experimental groups (Figure 3A-C). However, the decrease in the Emax of Ach was greater in controls and nor-NOHA-treated AIA rats (-45 ± 12% and -50 ± 6%, respectively) compared to untreated AIA group (-32 ± 6%). This result demonstrated that NOS activation is blunted in the case of arthritis and restored after nor-NOHA treatment. Conversely, the selective inducible NOS inhibitor 1400 W did not affect the response to Ach, whatever the group (data not shown). Because the role of EDHF is of critical importance for endothelial function, when NO production is compromised [31], we investigated the effect of nor-NOHA on EDHF production by incubating the rings with apamin and charybdotoxin. In favor of decreased EDHF production in arthritis, the combination of apamin/charybdotoxin significantly decreased the effect of Ach in control rats (Figure 3D) but not in AIA rats (Figure 3E) suggesting that the EDHF-mediated compensatory vasodilator system is lacking in arthritis. Nor-NOHA fully restored the EDHF component of Ach-induced relaxation in AIA rats (Figure 3F). To determine the role of prostanoids in endothelial dysfunction in AIA, aortic rings were first incubated with the non-selective COX inhibitor indometacin. While indometacin did not modify the relaxation to Ach in the control group (Figure 4A), it significantly improved vasodilation in untreated AIA rats (Figure 4B), indicating a deleterious role of COX activation in arthritis-induced endothelial dysfunction. More precisely, as a reflection of a deleterious role of the overactivation of COX-2, TX synthase and PGI_2 _synthase in endothelial dysfunction, the same patterns were observed after incubation with NS398 (Figure 4A), furegrelate and tranylcypromine, respectively, in controls (Figure 4B) and in AIA rats (Figure 4C, D). The treatment with nor-NOHA normalized the effect of these prostanoids inhibitors to that of control rats (Figures 4E, F).

**Figure 5:**
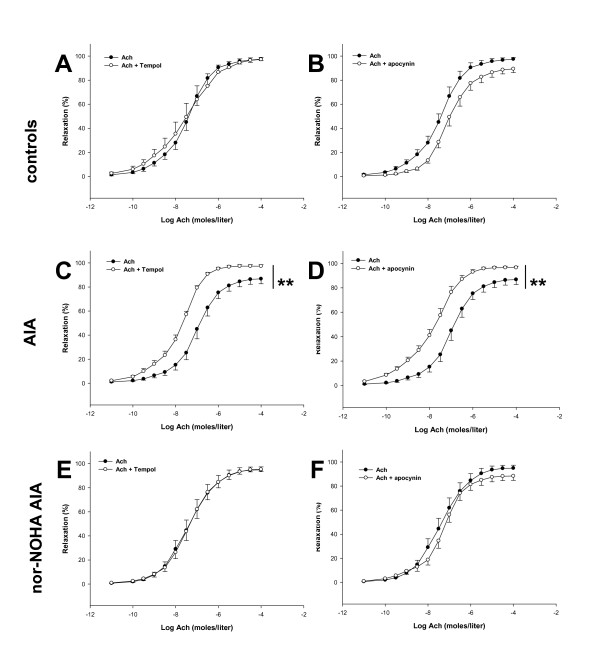
**Effect of Tempol and apocynin on vasodilation response to ACh**. Experiments were performed on aortic rings isolated from control rats (**A, B**), AIA rats (**C, D**) and nor-NOHA AIA rats (**E, F**) 21 days after the onset of arthritis. Cumulative concentration-responses curves with Ach were obtained after a 60-minute incubation period with Tempol at 10^-4 ^moles/liter (**A, C, E**) or with apocynin at 3 × 10^-4 ^moles/liter (**B, D, F**). Values are the mean ± SEM from 8 to 12 rings. ** = *P *< 0.01.

To assess whether the beneficial effects of nor-NOHA were linked to a decrease in O_2_^-. ^production in the aorta, rings were incubated with the SOD mimetic Tempol and with the NADPH oxidase inhibitor apocynin. Although neither Tempol nor apocynin altered relaxation to Ach in control group (Figure 5A, B), they significantly improved vasodilation in untreated AIA rats (Figure 5C, D), thus indicating that overproduction of O_2_^-. ^as well as overactivation of NADPH oxidase contribute to endothelial dysfunction in arthritis, as previously demonstrated [4]. After treatment with nor-NOHA, the Ach-induced relaxation remained unaffected by both compounds suggesting a normalization of O_2_^-. ^production and NADPH oxidase activity (Figure 5E, F).

## Discussion

Regarding how important is the prevention of cardiovascular events in RA, the understanding of the mechanisms associated to endothelial dysfunction, as well as the identification of new relevant treatments are of particular importance. The present study investigated for the first time the effect of a curative treatment with an arginase inhibitor (nor-NOHA) on vascular function of AIA rats. Our results demonstrated that this treatment improves endothelial function via an increase in NOS activity and EDHF production, a decrease in COX-2, TX synthase and PGI_2 _synthase activities as well as a decrease in O_2_^-. ^production. However, our data did not argue for a role of arginase in the chronic phase of inflammatory joint damages in the AIA model.

In accordance with previous studies [3-5,32-34], our data showed impaired endothelial function in AIA rats. To ascertain that the abnormal response of vessels from AIA rats to Ach was not due to decreased response of vascular smooth muscle cells (VSMCs) to NO, we demonstrated that the relaxing effect of the NO donor SNP is not impaired in AIA rats, in agreement with recent studies in rats [4,6,35] as well as in RA patients [36-38]. Likewise, to eliminate the contribution of an abnormal response of VSMCs to endothelium-derived vasoconstrictive mediators, we demonstrated that the effects of NE, ANG-II and ET-1 are unaltered in AIA rats. To understand the mechanisms involved in the effects of nor-NOHA, and because arginase and endothelial NOS share the same substrate L-arginine, aortic rings were first incubated with the non-selective NOS inhibitor L-NAME. Our data showed that the treatment with nor-NOHA significantly restored the decreased NOS activity observed in AIA rats. This result argues for the contribution of increased vascular arginase activity to NO deficiency in AIA rats, and demonstrated that *in vivo *arginase inhibition restores the equilibrium between NOS and arginase pathways, as previously identified in animal models of cardiovascular diseases, such as hypertension [10,11], atherosclerosis [13] or ageing [14]. Another new finding is the lack of effect of the selective iNOS inhibitor 1400 W on endothelial dysfunction in AIA. Data on vascular iNOS expression in AIA are controversial since the expression was found to be unchanged [4] or increased [35]. Our data showing the lack of effect of 1400 W on Ach-induced vasodilation, whatever the group of rats, did not argue for a contribution of the iNOS pathway in the impaired endothelial function associated to RA. This result contrasts with that of a study conducted in a small cohort of RA patients suggesting a deleterious role of increased vascular iNOS expression in vascular dysfunction [36]. However, in this study, the iNOS inhibitor used was aminoguanidine, which has a wide range of pharmacological actions besides iNOS inhibition including antioxidant effects [39], and it cannot be excluded that this latter property accounted for its vascular effect. Collectively, these data emphasize the need for further studies to define the role of iNOS in RA-associated endothelial dysfunction.

The COX pathway plays an important role in endothelial function. COX-1 is expressed constitutively and is usually abundant in all animal and human endothelial cells, whereas endothelial COX-2 is induced mainly during inflammatory response [40]. Physiologically, COX synthesizes both vasorelaxant prostanoids, such as PGI_2 _and vasoconstrictive prostanoids, such as TXA_2_. On the basis of the effects observed for indometacin and the preferential COX-2 inhibitor NS-398, the present study demonstrates for the first time that COX-2 activation contributes to endothelial dysfunction in aorta of AIA rats. Our result is consistent with the results of a recent study showing that vascular COX-2 expression was overexpressed in rabbits with both chronic AIA and atherosclerosis [41]. Although the contribution of COX-2 and TX synthase overactivation to endothelial dysfunction has been already demonstrated in animal models of cardiovascular diseases [40], the negative role of PGI_2 _is more surprising. In fact, our results confirm the janus face of PGI_2 _and suggest that, as described in spontaneously hypertensive rats (SHR) [42], PGI_2 _can induce vasoconstriction in AIA rats. Importantly, nor-NOHA treatment totally normalized the COX-pathway dysfunction in AIA rats. These data are in accordance with a previous study conducted in SHR in which nor-NOHA treatment was able to improve the COX-dependent component of Ach by decreasing vascular COX-2 expression [11].

Despite the ongoing debate of the molecular identity and signalling pathways, the contribution of endothelium-derived hyperpolarizing factor(s) (EDHFs) to the endothelium-dependent relaxation is also considered as an important feature of normal endothelium function [31]. EDHF has been demonstrated unequivocally in various blood vessels from different species, including human [43]. The acronym 'EDHF' is applied to a factor inducing vascular relaxation in the presence of COX plus NOS inhibitors and which is inhibited by charybdotoxin + apamin but unaffected by iberiotoxin + apamin. Animal studies have identified several mediators that might act as EDHF, such as K^+^, cytochrome P450 metabolites, lipoxygenase products, NO itself, H_2_O_2_, cyclic adenosine monophosphate, C-type natriuretic peptide and electrical coupling through myoendothelial gap junctions [43,44]. Whatever the mediator, EDHF induces a potassium-mediated event associated to a reduction in intracellular K^+ ^in vascular smooth muscle [45]. Experimental evidence indicates that EDHF action is of critical importance for endothelial function when NO production is compromised [31]. Our data showed for the first time that EDHF production is impaired in AIA rats, suggesting that the EDHF-mediated compensatory dilator system is lacking in RA. Of interest, our data reported that arginase inhibition restored the EDHF contribution to that of control rats. Because of the existence of a cross-talk between NO and EDHF pathways [31], it can be hypothesized that the improvement of NO production by nor-NOHA contributes to the recovery of EDHF production in AIA treated rats. However, albeit non-elucidated, a direct effect of nor-NOHA on EDHF production cannot be excluded.

Previous reports suggested that the overproduction of O_2_^-. ^contributes to endothelial dysfunction in AIA rats [4,5]. Consistent with these findings, the present study demonstrates the improvement of ACh-induced vasorelaxation with a SOD mimetic. In the AIA model, previous data reported that statins [5] as well as ANG-II receptor antagonists [6] were able to decrease aortic O_2_^-. ^overproduction. In the present study, we provide evidence that this beneficial effect can also be obtained with an arginase inhibitor. It is worth noting that recent reports reported that statins inhibit arginase activity [46,47] and that blockade of ANG-II AT1 receptors prevents the ANG II-induced elevation of arginase activity and expression [48]. Although speculative, we hypothesize that arginase could be the downstream common effector of these two types of drugs. Because the deficiency of L-arginine causes a NOS uncoupling leading to O_2_^-. ^production [40], the beneficial effect of arginase inhibition on O_2_^-. ^production might be due to the decrease in vascular eNOS uncoupling, as recently demonstrated in the vasculature of aged rats treated with the arginase inhibitor 2S-amino-6-boronohexanoic acid [49]. Additionally, since COX-2 was found to contribute to O_2_^- ^production [40], the nor-NOHA-induced decrease in COX-2 activity might also contribute to this effect. Moreover, our results confirmed the deleterious impact of increased NADPH oxidase activity to endothelial function in AIA [4] and demonstrated that nor-NOHA reverses this phenomenon. Whether arginase inhibition can directly reduce NADPH oxidase activity/expression is currently unknown but a recent study showing that NADPH oxidase inhibition reduced arginase upregulation in retinal cells [50] suggests an interplay between the two enzymes.

Although there is now ample evidence that RA is associated with endothelial dysfunction [1], several issues remain unresolved concerning the potential contribution of disease activity and classical cardiovascular risk factors. In addition, whether endothelial dysfunction occurs before the onset of RA or is a consequence of the disease is still a matter of debate. The presence of endothelial dysfunction in the very early stages of RA was recently demonstrated and was not explained by differences in disease activity and inflammatory markers [51]. Conversely, no impairment of endothelial function was observed in patients with < 7 years' disease duration whereas longer disease duration (> 14 years) was associated with severe endothelial dysfunction [52]. Others showed that endothelial dysfunction reflects enhanced inflammatory disease activity [38,53]. Moreover, on the observation that elevated inflammatory molecules are associated with increased risk of cardiovascular diseases in the general population, it has been speculated that RA-related inflammation might contribute to endothelial dysfunction [1]. However, as emphasized in a recent review [1], there is surprisingly no direct evidence supporting such an association between systemic inflammation and vascular dysfunction in RA patients. Therefore, two hypotheses might be formulated to explain how a treatment can improve endothelial function in RA. First, the benefits might be secondary to decreased systemic and/or vascular inflammation and disease activity. Second, the treatment may act directly on endothelial homeostasis independently of inflammation or disease activity. In our study, we showed that nor-NOHA treatment did not modify disease severity assessed by clinical, histological and radiological parameters, whereas it fully reversed endothelial dysfunction in AIA rats. These results demonstrate that the reduction of endothelial dysfunction is possible even though articular parameters are not improved and that endothelial dysfunction is not the consequence of the disease, at least in the chronic phase of the AIA model. These data are in keeping with a recent report showing that an angiotensin-converting enzyme (ACE) inhibitor improved endothelial dysfunction without any change in disease activity in RA patients [54]. In this study, treatment with ramipril markedly improved endothelial function, although it modestly reduced circulating cytokines, indicating that other mechanisms than the reduction of vascular inflammation are likely involved in the beneficial effects of the ACE inhibitor. Likewise, our data showing that plasma IL-6 levels did not correlate with endothelial dysfunction strongly suggest that the benefits provided by nor-NOHA are related to the direct modulation of endothelium-derived vasorelaxant pathways rather than an anti-inflammatory effect. In addition, our data also suggest that arginase is not upregulated at the articular level and/or not involved in the evolution of the disease between the Day 13 and the Day 34 post-immunization, that is, during the chronic phase of AIA. These data are in agreement with a recent study conducted on arthritic mice showing that arginase gene expression in synovial tissue increased during acute inflammation but not during chronic inflammation [55]. Further studies will be necessary to explore the role of arginase at the articular level in RA.

With the aim to identify clinically relevant peripheral markers of endothelial dysfunction in RA, we measured plasma VEGF levels in control and AIA rats. Although increased VEGF level is considered as a marker of endothelial dysfunction in hypercholesterolemia, atherosclerosis or hypertension [28], whether VEGF is a marker of endothelial dysfunction in RA is not known. Previous studies conducted in RA patients reported elevated VEGF levels in serum, which were related to angiogenesis of synovial pannus but not with endothelial dysfunction [56]. In the present study, we showed that VEGF levels are increased in AIA rats, and that nor-NOHA treatment decreased VEGF levels, whereas arthritis severity was unchanged. Importantly, we identified the existence of a negative correlation between VEGF levels and endothelial function, suggesting for the first time that high plasma VEGF levels might reflect endothelial dysfunction in RA. The mechanisms explaining the decrease in VEGF levels after arginase inhibition were not determined in the present study but might rely on decreased IL-6 production since VEGF synthesis is IL-6 dependent [57].

## Conclusions

In conclusion, the present study on AIA rats gives important new data for the management of endothelial dysfunction in RA. First, it identifies decreased EDHF production and increased COX-2 activity as new pathophysiological mechanisms of endothelial dysfunction besides decreased NOS activity and O_2_^-. ^overproduction, thus providing new potential targets for the treatment of this cardiovascular risk factor in RA. Second, it demonstrates that the treatment with an arginase inhibitor is a promising and efficient approach for reversing endothelial dysfunction in RA, as originally suggested in animal models of "traditional" cardiovascular diseases [58]. Although these data obtained in AIA rats have to be confirmed by clinical studies, our data strongly suggest that arginase inhibition has the potential as a novel add-on therapy in the treatment of RA.

## Abbreviations

ACE: angiotensin-converting enzyme; Ach: acetylcholine; AIA: arthritis induced adjuvant; ANG-II: angiotensin-II; ANOVA: one-way analysis of variance; BH4: tetrahydrobiopterin; COX: cyclooxygenase; ED: endothelial dysfunction; EDHF: endothelium-derived hyperpolarizing factor; ELISA: enzyme-linked immunosorbent assay; Emax values: The values of maximal relaxation; eNOS: endothelial Nitric Oxide Synthase; ET-1: endothelin-1; HES: hematoxylin-eosin and safranin; IL: interleukin; iNOS: induced Nitric Oxide Synthase; L-NAME: N_W_-nitro-L-arginine methyl ester; MB: *Mycobacterium butyricum*; NADPH: the nicotinamide adenine dinucleotide phosphate; NE: norepinephrine; NO: nitric oxide; nor-NOHA: N_w_-hydroxy-nor-L-arginine; NOS: nitric oxide synthase; O_2_^-.^: superoxide anions; PGI_2_: prostacyclin; RA: rheumatoid arthritis; SEM: standard error of the mean; SHR: spontaneously hypertensive rats; SNP: sodium nitroprussiate SOD: superoxide dismutase mimetic; TNFα: tumor necrosis factor α; TXA_2_: thromboxane A_2_; VEGF: vascular endothelial growth factor; VSMCs: vascular smooth muscle cells

## Competing interests

The authors declare that they have no competing interests.

## Authors' contributions

CP conceived of the study, and participated in its design and coordination, and helped to draft the manuscript. He also carried out histology, radiology, vascular function analysis, treatment of animals and ELISA kits. AB conceived of the study, participated in its design and coordination, and helped to draft the manuscript. BK carried out the histology analysis and helped to draft the manuscript. DW conceived of the study, participated in the design of the study and coordination, and helped to draft the manuscript. CD conceived of the study, and participated in its design and coordination, helped to draft the manuscript and performed the statistical analysis. All authors read and approved the final manuscript.

## Supplementary Material

Additional file 1 **Time-course of clinical arthritic score in AIA and nor-NOHA-treated AIA rats**. AIA rats were monitored seven days per week in a blinded fashion for clinical signs of arthritis. Day 0 = day of injection with *Mycobacterium butyricum*. The nor-NOHA treatment was started with the first clinical signs of arthritis. *n *= 20 rats per group. NS, Non-significant. Click here for file

Additional file 2 **Regression analysis between plasma VEGF and endothelial dysfunction**. A significant positive correlation was found between plasma VEGF levels and endothelial dysfunction as attested by the negative correlation identified between the Emax of Ach and the plasma VEGF levels of control, AIA and nor-NOHA-treated AIA rats (*n *= 8 rats per group). Click here for file
