# Supplementary material for: Treatment with the arginase inhibitor Nw-hydroxy-nor-L-arginine restores endothelial function in rat adjuvant-induced arthritis
Source: Arthritis Res Ther. 2012 May 30;14(3):R130. doi: 10.1186/ar3860 (PMC3446511; doi:10.1186/ar3860)
Supplement: Supplementary file 1 [file ar3860-S1.DOC]

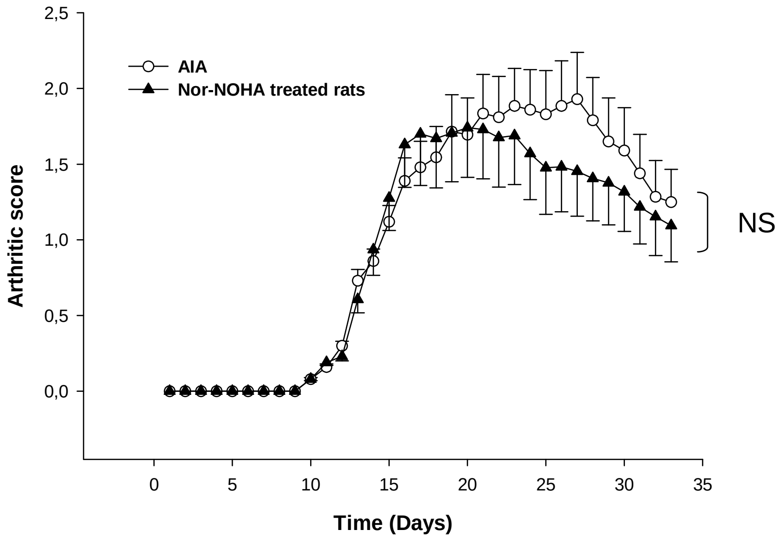

AIA
Nor-NOHA treated rats
Arthritic score
Time (Days)
2,5
2,0
1,5
1,0
0,5
0,0
0
5
10
15
20
25
30
35
NS
