# Supplementary material for: Treatment with the arginase inhibitor Nw-hydroxy-nor-L-arginine restores endothelial function in rat adjuvant-induced arthritis
Source: Arthritis Res Ther. 2012 May 30;14(3):R130. doi: 10.1186/ar3860 (PMC3446511; doi:10.1186/ar3860)
Supplement: Supplementary file 2 [file ar3860-S2.DOC]

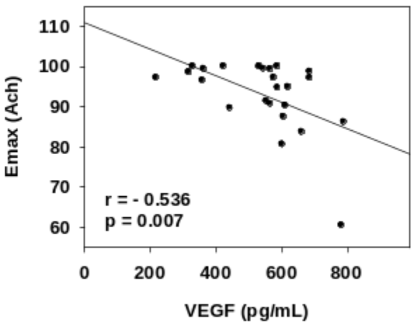
110
100
90
80
70
60
Emax (Ach)
r = - 0.536
p = 0.007
0
200
400
600
800
VEGF (pg/mL)
